# Older Latinos’ Perceptions of the Caregiving Experience

**DOI:** 10.1093/geroni/igab046.2924

**Published:** 2021-12-17

**Authors:** Michelle Jaldin, Guilherme Balbim, Isabela Marques, David Marquez

**Affiliations:** 1 University of Illinois at Chicago, Chicago, Illinois, United States; 2 University of British Columbia, Vancouver, British Columbia, Canada; 3 Universidade de Sao Paulo, Sao Paulo, Sao Paulo, Brazil; 4 University of Illinois at Chicago & Rush University, University of Illinois at Chicago, Illinois, United States

## Abstract

There has been a rapid growth of Latinos age 65 and older in the United States and the population is projected to grow to 21.5 million by 2060. Latinos with Alzheimer’s disease is expected to increase 832% by 2060. Caregiving for adults with Alzheimer’s Disease and Related Dementias (ADRD) is physically, emotionally, and financially demanding, and has significant implications for caregivers’ health, personal and social life, and overall well-being. This study aimed to describe the perceived experiences of middle-aged and older Latino who were primary caregivers of relatives with ADRD. We conducted semi-structured interviews with Latino caregivers to examine their perceived experiences of providing care for a relative with ADRD. Interviews were conducted in English and Spanish and were transcribed, translated into English when needed, and coded. We conducted direct content analysis. Participants were aged 50 to 75 years (n = 16), the majority were female (n = 12), and majority were caring for either their parent or spouse. We identified six reoccurring themes in the Latino caregiving experiences: (1) caregiver burden; (2) dealing with care recipient; (3) coping strategies; (4) social support; (5) cultural values; and (6) knowledge about services. The identified themes showed that Latino caregivers need support from their family and friends for caregiving. Latino family’s structure plays an important role in caregiving experience. These themes are important to consider in future interventions that aim to reduce caregiver burden in Latinos as they influence the overall well-being of the caregiver.

